# A study on the optimal condition of ground truth area for liver tumor detection in ultrasound images using deep learning

**DOI:** 10.1007/s10396-023-01301-2

**Published:** 2023-04-04

**Authors:** Taisei Tosaki, Makoto Yamakawa, Tsuyoshi Shiina

**Affiliations:** 1 Graduate School of Medicine, Kyoto University, Kyoto, Japan; 2 SIT Research Laboratories, Shibaura Institute of Technology, 3-7-5 Toyosu, Koto-ku, Tokyo, 135-8548 Japan

**Keywords:** Artificial intelligence, Deep learning, Liver tumor detection, Ultrasound images

## Abstract

**Purpose:**

In recent years, efforts to apply artificial intelligence (AI) to the medical field have been growing. In general, a vast amount of high-quality training data is necessary to make great AI. For tumor detection AI, annotation quality is important. In diagnosis and detection of tumors using ultrasound images, humans use not only the tumor area but also the surrounding information, such as the back echo of the tumor. Therefore, we investigated changes in detection accuracy when changing the size of the region of interest (ROI, ground truth area) relative to liver tumors in the training data for the detection AI.

**Methods:**

We defined D/L as the ratio of the maximum diameter (D) of the liver tumor to the ROI size (L). We created training data by changing the D/L value, and performed learning and testing with YOLOv3.

**Results:**

Our results showed that the detection accuracy was highest when the training data were created with a D/L ratio between 0.8 and 1.0. In other words, it was found that the detection accuracy was improved by setting the ground true bounding box for detection AI training to be in contact with the tumor or slightly larger. We also found that when the D/L ratio was distributed in the training data, the wider the distribution, the lower the detection accuracy.

**Conclusions:**

Therefore, we recommend that the detector be trained with the D/L value close to a certain value between 0.8 and 1.0 for liver tumor detection from ultrasound images.

## Introduction

In recent years, efforts to apply artificial intelligence (AI) to the medical field have been growing [1]. Researchers expect AI to make up for the shortage of medical specialists and technologists by enabling them to screen people or follow-up on chronic diseases more quickly and efficiently [2–5]. In 2020, malignant tumors were the most common cause of Japanese death [6], with liver cancer being the fifth highest cause of cancer death [7]. Therefore, automatically detecting liver cancer by means of deep learning will play a pivotal role in early diagnosis and treatment.

In general, researchers need a vast amount of high-quality training data to make great AI. Recently, the Japan Society of Ultrasonics in Medicine (JSUM) supported by the Japan Agency for Medical Research and Development (AMED) has been collecting ultrasound images and constructing a large-scale database. When using this database, the number of images does not matter. However, the registered size of the square region of interest (ROI), i.e., the ground truth area for detection, relative to the size of the tumor varies in this database, because many doctors decide the size of the ROI based on the individual criterion when they register images to the database. Thus, the quality of annotation is the crucial issue to make good AI for tumor detection. In the database described above, the diagnosis name labels are comprehensively determined by pathological, MRI, and ultrasonic diagnosis, so we think that the quality of labeling is sufficiently high.

In the field of ultrasound image-based tumor detection using deep learning, some researchers have already reported the detection of breast tumors [8, 9] or thyroid nodules [10–12] using deep learning. However, there are few reports on detection of liver tumors based on ultrasound images using deep learning, although some studies on the classification of liver tumors based on ultrasound images using deep learning have been reported [13–19].

Furthermore, in terms of object detection, Xu et al. have shown that inaccurate labeling, translation of the center coordinates of the ROI, and the ratio of noise at the ROI affect the detection accuracy [20]. However, they have not evaluated the effect of ROI size variation relative to objects in the training data set.

On the other hand, in terms of classification, Yamakawa et al. have already evaluated the effect of ROI size variation relative to the liver tumor on ultrasound images using deep learning [13]. According to this research, a model trained with an appropriately large ROI relative to the tumor was more accurate in classification than a model trained with a ROI that touched the tumor. Therefore, a good classification AI model can be achieved when it learns not only the features inside the tumor but also the features around it. In fact, hepatic cysts and other liver tumors sometimes have a high-intensity line or a shadow in the posterior part of the tumor, which serve as the reference for human liver tumor detection. Based on the above, we investigated the effect on the detection accuracy when the ROI size varied.

## Methods

### Data

**Fig. 1:**
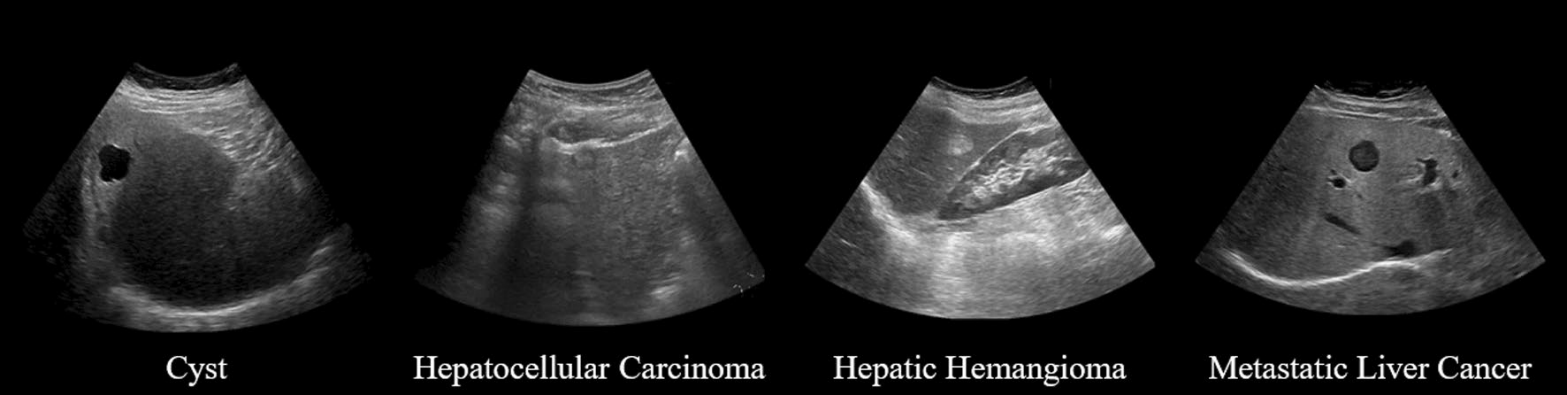
Example images of each type of liver tumor

We used ultrasound images of liver tumors collected by JSUM with the support of AMED. These images were collected by 11 hospitals in Japan. Therefore, these images were taken with ultrasound diagnostic equipment and probes from various manufacturers, and the image parameters also differed depending on the hospital and patient. In this study, we reconfigured the accurate tumor ROI for data collected as of August 2019. This data set includes 3245 cyst images (925 cases), 1364 hepatocellular carcinoma (HCC) images (304 cases), 1786 hepatic hemangioma (hema) images (562 cases), and 1212 metastatic liver cancer (meta) images (205 cases), as shown in Table 1. Figure 1 shows an example of each liver tumor image. In addition, since all the data used in this paper are still images, there are images that the doctor judged to be suitable for diagnosis, and data of extremely poor quality are not included.

**Table 1:** Data on ultrasound images of liver tumors used in this study

		Images	Cases
Type	Cyst	3245	925
	Hepatocellular carcinoma	1364	304
	Hepatic hemangioma	1786	562
	Metastatic liver cancer	1212	205
Total		7607	1996

### The condition of ground truth area

Based on the liver tumor center coordinates and size information registered in the database, we created the training data sets under different conditions, so that the size of the square ROI (the ground truth area for detection) relative to the tumor size was constant. Tumor center coordinates were not changed when generating the training data sets. Here, we used the center of the smallest circle circumscribing the tumor as the tumor center coordinates.

**Fig. 2:**
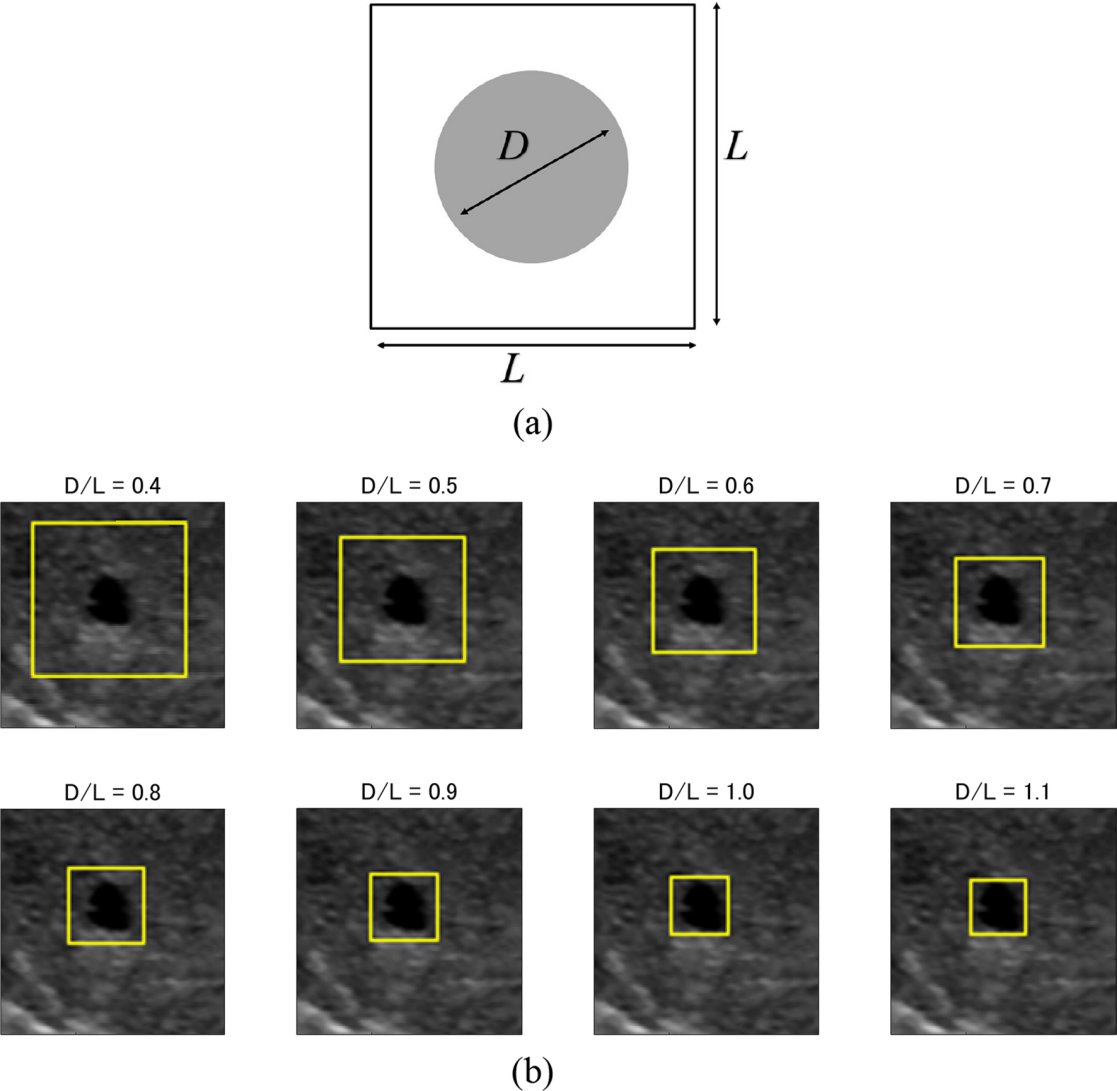
Definition of D/L and the ROI of each D/L. **a** Definition of D/L. **b** ROI of each D/L (yellow box)

As the quantitative metric for the ROI size compared with the tumor size, we used the D/L ratio, which was defined as the maximum diameter of the tumor divided by the ROI size, as shown in Fig. 2a.

We created the training data sets under the condition that the D/L ratio was 0.4–1.1. Figure 2b shows examples of ROIs with different D/L ratios. As shown in Fig. 2b, the ROI is directly in contact with the tumor when the D/L ratio equals 1. In addition, the ROI is larger than the tumor when the D/L ratio is less than 1, and in contrast, the ROI is smaller than the tumor when the D/L ratio is more than 1.

### Preprocessing and data augmentation

We created three data sets consisting of all images divided into training and test data sets at a ratio of 8:2, as shown in Table 2. Although we randomly divided all images into training and test data sets, we generated three sets of data sets for more accurate evaluation. Since the ultrasound images from the same case were similar, we divided all images by case to avoid having images from the same case in both the training and test data sets. In addition, there is no duplication of test data in the three data sets. Each training data set was then doubled by the horizontal flipping to acquire sufficient training images. In addition, to prevent the ROI from becoming larger than the image size when enlarging the ROI, we added a background margin around the ultrasound images. We set the margin size to be 25 pixels (in the resized image). We eliminated training images, where the ROI coordinates exceeded the image size even after adding the margin. The number of images we eliminated is shown in Table 3. Finally, we resized all images to 448 × 448 pixels, which is the input size of YOLOv3. In other words, the image input into YOLOv3 is the entire ultrasound image resized to 448 × 448 pixels. In the resizing process, we used the linear interpolation method to reduce the image size.

**Table 2:** Three data sets used in this study

(a) Data set 1		Training	Test
Type	Cyst	2578	667
	Hepatocellular carcinoma	1086	278
	Hepatic hemangioma	1376	410
	Metastatic liver cancer	1017	195
Total		6057	1550

(b) Data set 2		Training	Test
Type	Cyst	2562	683
	Hepatocellular carcinoma	1064	300
	Hepatic hemangioma	1437	349
	Metastatic liver cancer	1017	195
Total		6080	1527

(c) Data set 3		Training	Test
Type	Cyst	2608	637
	Hepatocellular carcinoma	1059	305
	Hepatic hemangioma	1432	354
	Metastatic liver cancer	958	254
Total		6057	1550

**Table 3:** Number of images where the ROI exceeded the image area

D/L	Data set 1	Data set 2	Data set 3
0.4	489	603	498
0.5	157	201	99
0.6	32	38	27
0.7	1	1	5
0.8	0	0	4
0.9	0	0	0
1.0	0	0	0
1.1	0	0	0

### YOLOv3

There are two main kinds of models for object detection: a one-stage model such as the YOLO series [21–23] and SSD [24] and a two-stage model such as the Fast R-CNN [25] and Faster R-CNN [26]. A one-stage model has a high detection speed, while a two-stage model has a low detection speed [27]. In this study, we used the YOLOv3 model [23], because it is a one-stage model that can detect objects in real time. The YOLOv3 model is a multi-scale detector that detects tumors from three different scale feature maps. These feature maps allow YOLOv3 to find objects of various sizes with high accuracy in real time. The input to YOLOv3 in this research was an image resized to 448 × 448 pixels.

We executed one-class detection, i.e., detecting and classifying “tumor” or not. We chose one-class detection for the following two reasons. First, in a preliminary study, one-class detection showed higher detection capability than that of four-class detection (four-class means cyst, HCC, hepatic hemangioma, and metastatic liver cancer). Second, both detecting tumors and classifying them at the same time are not appropriate tasks for a detection model. Therefore, we are considering a computer-aided diagnosis system that first detects tumors with a detection model and then classifies tumor types with a classification model.

The base network of YOLOv3 was darknet-53, the initial learning rate was 1e-04, the mini-batch size was 4, and the max epochs were 40. The output was an estimated square box covering the tumor and a confidence score. We processed images three times under each D/L condition to precisely evaluate the change in evaluation metrics.

### Evaluation method

Unlike training data sets, we fixed the D/L condition at 1.0 when setting the ground truth area for evaluating results on the test data set. For example, a small D/L and a large ground truth area on the test data set can lead to overestimation. Therefore, to accurately evaluate the results of each D/L, we fixed the D/L to 1.0 in the test data set.

The test data set included ultrasound images that had multiple tumors. However, there was only one annotated tumor in the JSUM database. Therefore, when the YOLOv3 model detected multiple objects in a single image, we evaluated the detection box that had the highest confidence score in the positive IoU ratio between the predicted and ground truth bounding boxes. If there were only detection boxes with an IoU ratio of 0, we evaluated the detection box with the highest confidence score. Therefore, it is possible that a detection box determined as false positive (FP) is correctly detecting tumors that are not registered. Still, true positive (TP) and false negative (FN) can be evaluated correctly. Therefore, there is no problem with the evaluation for the purpose of this study even if the evaluation is only for the above detection box.

**Fig. 3:**
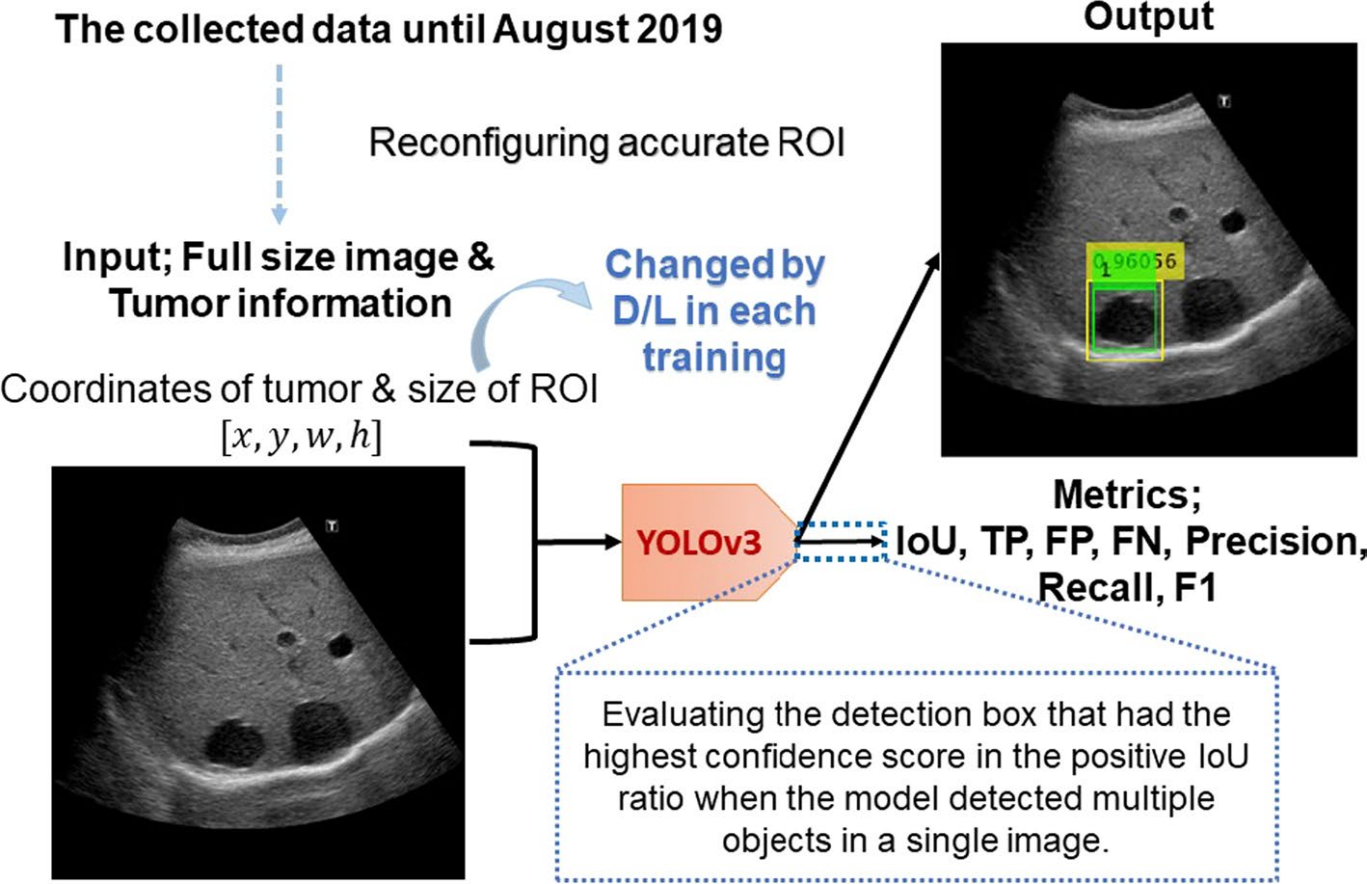
Rough flow of training YOLOv3 and evaluation. $(x,y)$ are the coordinates of the upper-left corner of the ground truth box, and $(w,h)$ are the size of the ground truth box. The ground truth box is green, and the detection box is yellow in the output image. The number above the detection box represents the detection confidence score

The rough flow of training and evaluating the YOLOv3 model is shown in Fig. 3. In this study, we used the IoU, which is used in many object detection studies, when evaluating the predicted bounding boxes. In the following, we show the definition of the evaluation metrics we used.

1. IoU (intersection of union)

IoU is defined as follows:

1 $$\mathrm{IoU}= \frac{Area\, of\, {B}_{p}\cap {B}_{gt}}{Area\, of\, {B}_{p}\cup {B}_{gt}},$$

where ${B}_{p}$ is the predicted bounding box and ${B}_{gt}$ is the ground true bounding box.

2. True positive (TP)

The number of images where the IoU ratio was higher than the IoU threshold.

3. False positive (FP)

The number of images where the IoU ratio was lower than the IoU threshold.

4. False negative (FN)

The number of images where the YOLOv3 model could not detect anything.

5. 2 $${\text{Precision}} = { }\frac{TP}{{TP + FP}}$$
6. 3 $$\mathrm{Recall}= \frac{TP}{TP+FN}$$
7. 4 $$\mathrm{F}1\mathrm{ Score}= \frac{2 \times Precision \times Recall}{Precision+Recall}$$

We report the maximum value of precision, recall, and F1 score and the minimum score of FN in the three times.

In addition, since there are databases in which the tumor ROI is not strictly set, we also used the above metrics to evaluate cases, where the D/L value in the training data was not fixed but distributed (in Section “Distribution range of D/L values”).

### How to decide the IoU threshold

As shown in Section “Evaluation method”, since the images with IoU above the threshold are correct answers (TP) and the images with IoU below the threshold are incorrect answers (FP, FN), setting the appropriate threshold value is essential when we evaluate each YOLOv3 model. However, previous studies on tumor detection in ultrasound images [8–12, 19] did not standardize the threshold and how to decide it. Therefore, we determined the threshold by discriminant analysis using the IoU distribution when D/L was 1.0 in each data set (Data 1–3). Here, we used the FN results as IoU = 0. Since the discriminant analysis method calculates the threshold that maximizes the inter-class variance of two classes divided by the threshold, we considered discriminant analysis to be an appropriate method for determining the threshold in this study. Discriminant analysis is also called Otsu’s method and is used to determine the threshold for binarizing an image. The IoU threshold calculated by discriminant analysis was 0.375 for all data sets. Therefore, we determined that the IoU threshold to calculate evaluation metrics was 0.375.

## Results

### Total evaluation metrics

**Fig. 4:**
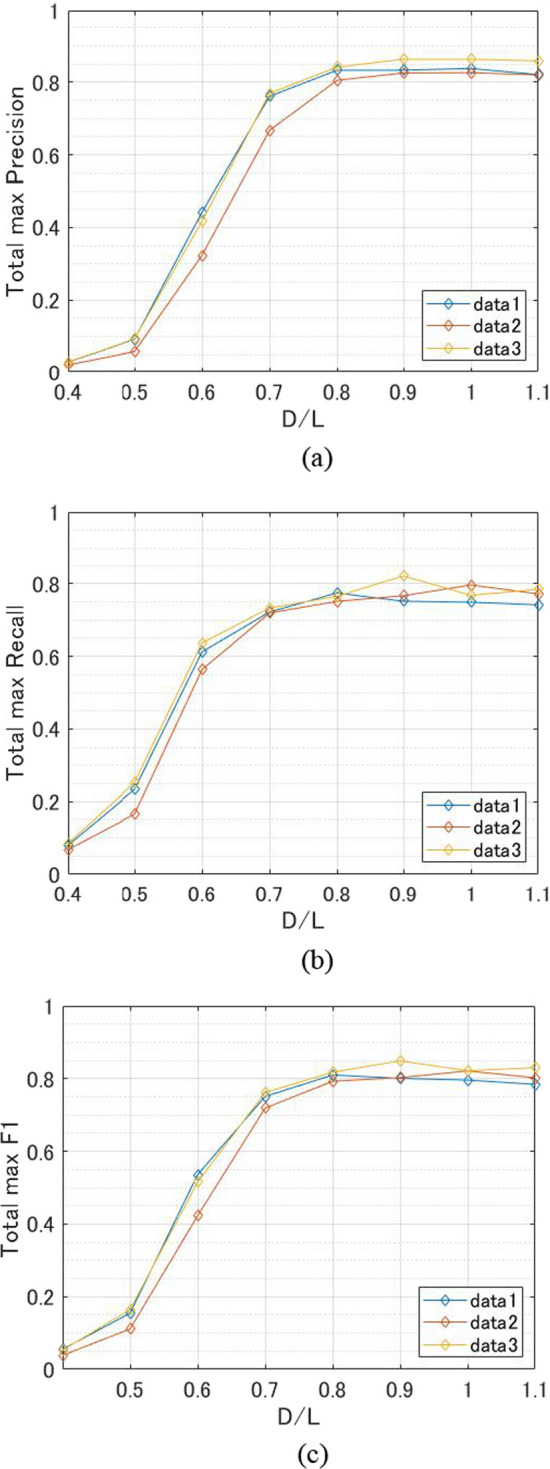
Results of total evaluation metrics. **a** Precision. **b** Recall. **c** F1

Figure 4 shows the results of total evaluation metrics obtained by changing the D/L value. Precision decreased when D/L was less than 0.7 and recall also decreased when D/L was less than 0.6. The precision showed less fluctuation when D/L was 0.8–1.1, while recall was highest when D/L was 0.8 (Data 1), 0.9 (Data 3), and 1.0 (Data 2). The maximum value of F1 was observed in Data 3 when D/L was 0.9, but in the other two data sets, there was no significant change when D/L was more than 0.8. When D/L was 0.4, precision, recall, and F1 score were close to 0.

### FN

**Fig. 5:**
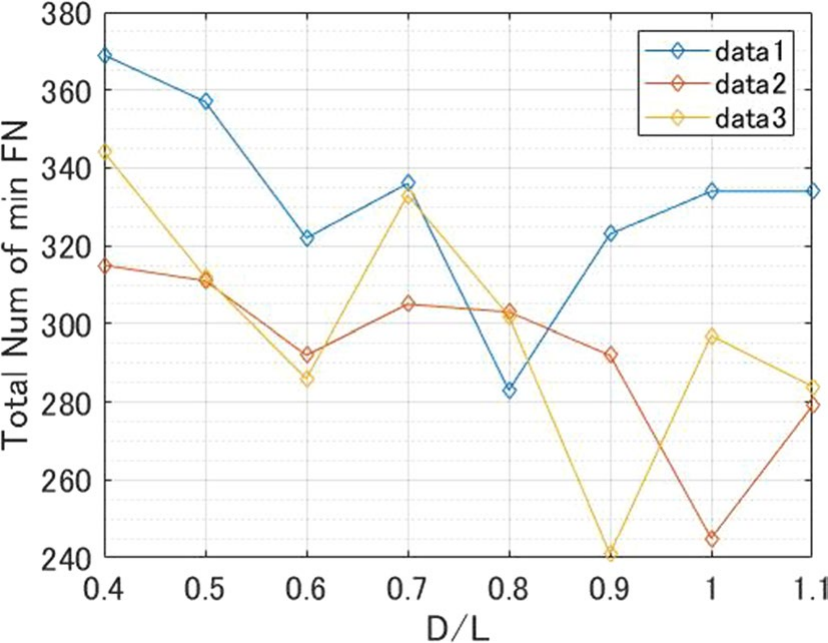
Result of total FN

FN indicates the number of images, where YOLOv3 did not output any detection box. Therefore, FN allows us to evaluate the threshold-independent performance of YOLOv3. The results are shown in Fig. 5. FN of Data 1 was lowest when D/L was 0.8, that of Data 2 was lowest when D/L was 1.0, and that of Data 3 was lowest when D/L was 0.9, corresponding to the values of D/L at which recall was highest. In addition, when D/L was 0.4, FN was the worst in all data sets.

### Examples of detection images

**Fig. 6:**
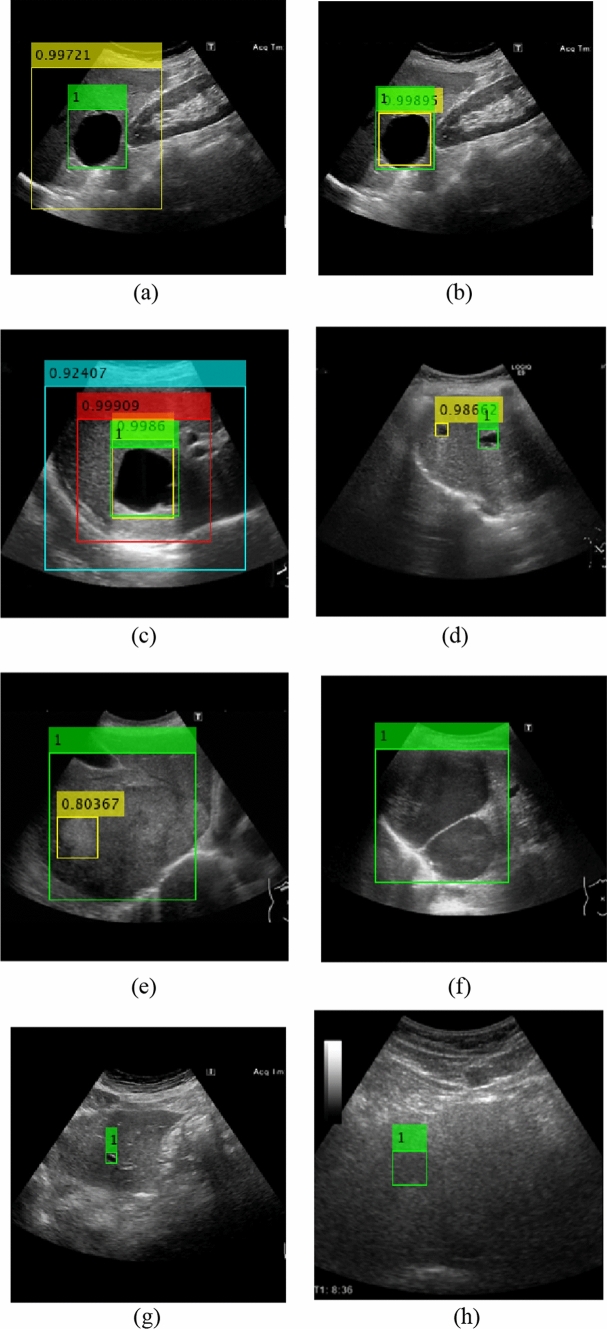
Examples of detection images. **a** D/L = 0.4. **b** D/L = 1.0. **c** Detection box with each D/L. **d** Detecting blood vessels (FP). **e** Detecting part of the bull’s eye pattern (FP). **f** Large tumor (FN). **g** Small tumor (FN). **h** Hard-to-recognize tumor (FN). The ground truth box is green (**a**–**h**), and the detection box is yellow (**a**), (**b**), (**d**), (**e**). **c** The detection boxes: D/L = 0.4 (cyan), 0.6 (red), 1.0 (yellow). Numbers above the boxes represent the detection confidence scores. A higher score indicates higher confidence in the detection

We show the detection results in Fig. 6. The numbers above the detection boxes in Fig. 6 represent the detection confidence scores. The detection box tended to be larger than the tumor as the D/L decreased-ROI increased (Fig. 6a). On the other hand, when D/L increased-ROI decreased, the detection box was often tangent to or slightly smaller than the tumor (Fig. 6b). In addition, there were few images in which the center coordinates of the detection box were far from the center coordinates of the ground truth ROI regardless of the D/L value (Fig. 6c).

As shown in Fig. 6d–h, there were some images that were detected incorrectly: detecting blood vessels in Fig. 6d and detecting only a part of the bullseye pattern in Fig. 6e. Examples of FN images were those in which the maximum diameter of the tumor was more than half the size of the input image (Fig. 6f), those in which the tumor size was fairly small (Fig. 6g), and those in which the tumor was difficult for humans to recognize (Fig. 6h).

## Discussion

### Precision, recall, and FN with different D/L

**Fig. 7:**
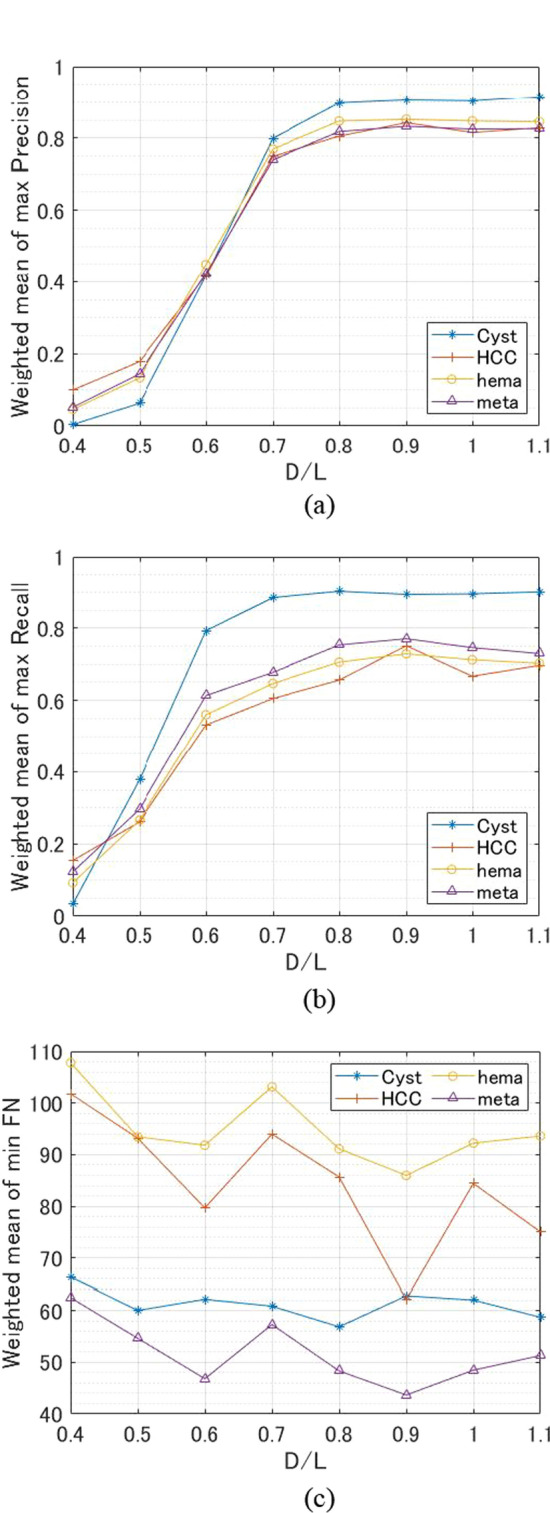
Results for each tumor type. **a** Precision. **b** Recall. **c** FN

Total precision decreased significantly when the D/L value was less than 0.7, and total recall and total FN were best when the D/L value was between 0.8 and 1.0. Below, we discuss whether the fluctuation of metrics with each D/L is due to the type of tumor. The results for precision, recall, and FN by tumor when D/L varied are shown in Fig. 7. Each graph displays the weighted average of the three data sets' maximum precision, recall, or minimum FN. Here, the weighted average represents the average value weighted by the number of test data included in each data set. In the case of precision and recall, cyst showed the highest values among the four tumor types when D/L was higher than 0.6. Precision, recall, and FN in the case of cyst fluctuated less than for other tumors when D/L was 0.7 or greater. Therefore, cyst is the tumor type that is least affected by D/L variation. On the contrary, HCC, which had the largest recall and the smallest FN when D/L was 0.9, is the tumor type that is most affected by variation in the D/L value.

Looking at the results for total precision, total recall, and total F1, since the amount of decrease in the evaluation metrics was small even when the D/L was increased from the D/L at the maximum evaluation metrics, information inside the tumor presumably has a greater influence than the information around the tumor. However, according to the results for total FN, the number of FN clearly increased when the D/L was increased from the D/L at the minimum number of FN. Therefore, information around the tumor is also considered important. In addition, the evaluation metrics did not change significantly between D/L of 1.0 and 1.1, suggesting that information near the tumor boundary is as important as information inside the tumor. However, even if the D/L is 1.1, part of the boundary is included in the ground truth area, so further verification is necessary in the future.

### Distribution range of D/L values

In this section, we discuss the case, where the D/L value is not fixed. Table 4 shows how each evaluation metric changed when we trained the YOLOv3 within several ranges of D/L values (0.6–1.0, 0.7–1.0, 0.8–1.0). The D/L values are uniformly distributed within the given D/L range. As shown in Table 4, the smaller the range of D/L values, the better the total evaluation metrics.

**Table 4:** Results when D/L values are distributed

	D/L	FN	Precision	Recall
Data set 1	0.6–1.0	360	0.722	0.705
	0.7–1.0	321	0.827	0.755
	0.8–1.0	**208**	**0.840**	**0.835**
	Constant (best D/L value)	283 (0.8)	0.837 (0.9)	0.774 (0.8)
Data set 2	0.6–1.0	340	0.648	0.685
	0.7–1.0	321	0.790	0.748
	0.8–1.0	308	**0.839**	0.765
	Constant (best D/L value)	**245 (1.0)**	0.826 (1.0)	**0.812 (1.0)**
Data set 3	0.6–1.0	348	0.735	0.713
	0.7–1.0	366	0.841	0.720
	0.8–1.0	289	**0.877**	0.790
	Constant (best D/L value)	**241 (0.9)**	0.863 (0.9)	**0.821 (0.9)**

Bold represents the best results for each dataset

To demonstrate the optimal D/L setting, this table shows results with distributed D/L values and best results with constant D/L values

Recall and FN were generally better with a fixed D/L than with a distributed D/L. However, precision was slightly better when the D/L was between 0.8 and 1.0.

Therefore, we suggest that the detector be trained with the D/L value close to a certain value within 0.8–1.0.

### Differences in optimal D/L values for detection and classification

In this study, we confirmed that setting the ground truth region with the D/L between 0.8 and 1.0 was optimal for liver tumor detection based on ultrasound images. On the other hand, in our previous study [13], we confirmed that an ROI with a D/L of 0.6 was optimal for classifying ultrasound images of liver tumors. In other words, liver tumor classification requires more information around the tumor as compared with detection. We believe that the reason for this difference is that detection in this study only determines whether a liver tumor is present or not, whereas classification must determine the liver tumor type. For example, when classifying HCC and metastatic liver cancer, the fibrosis state of the liver parenchyma (region around the tumor) is an important criterion. Therefore, we believe that liver tumor classification is more accurate with a larger peritumoral region as compared with detection.

## Conclusion

In this study, we examined how the detection capability and detection accuracy changed when the ROI size for the tumor (D/L) was varied.

Precision and recall decreased significantly when the D/L value was less than 0.7, and FN was lowest when the D/L value was 0.8, 0.9, or 1.0. In terms of tumors, the detection capability for cysts was constant with the change of D/L value, but it changed significantly in the other three tumors, especially in the case of HCC. When D/L values were distributed, almost all evaluation metrics including precision, recall, and FN became worse as the range of D/L values increased. Therefore, we consider that reducing the scatter of D/L values in training data improves the detection capability. We conclude that the D/L distribution should be kept between 0.8 and 1.0 for liver tumor detection based on ultrasound images.

In general, the quality of annotation in object detection includes two main categories: the label accuracy and the noisy annotation of the ROI. This noisy annotation generally means the gap between the center coordinates of the ROI and that of the target object. However, our research suggests that the ground truth ROI size for the target object is also one of the parameters of noisy annotation. In particular, the D/L condition and variation become a problem where we need both background information of the object and the features of the object itself for detection using deep learning. As for the D/L condition, we should pay attention to the following two parameters: the size of the D/L value itself and the distribution range of the D/L value. We improved the detection capability most when the D/L value was less than 1.0 and the range of D/L was also small. This criterion can also be used as a rough guide in other fields of ultrasound diagnosis. However, optimal conditions may vary for each tumor and organ of interest.

Future research should include training a robust model against variation of D/L values and developing a model to keep the D/L value constant in the database.

## Data Availability

The data that support the findings of this study are only available to the ethics committee-approved researchers and are not publicly available.
